# Does Breathing through the Mouth Injure the Teeth?

**Published:** 1863-03

**Authors:** Geo. F. Foote


### DOES BREATHING THROUGH THE MOUTH INJURE THE TEETH.

Read before the Mississippi Valley Dental Association.

BY GEO. F. FOOTE, M. D.

There are three sets of glands emptying saliva into the mouth, viz.: the parotid, through the duct of steno, opposite the second upper molars; the sub-maxillary, through the ducts of wharton, at the frenum of the tongue, and the sub-lingual through many openings between the tongue and the cuspid and bicuspids. The saliva from the first is most profuse, comprising about two-thirds of all secreted; more limpid than the rest, and seems designed in one of its functions at least, to supply moisture to the food. It is poured into the mouth during mastication and for some time after, and its quantity depends upon the dryness, and to some extent, upon the stimulating qualities of the food eaten. That from the second, or submaxiliary, comprises about $\frac{1}{20}$ of the whole, is more viscous and would seem to serve the purposes of lubricating the food so as to make deglutition easy, and according to some authors it has the power of converting starch into sugar, (which the former has not,) and thus forms an important function in the process of digestion. Animals that swallow their food whole, like birds and reptiles, have no parotid glands, but have the sub-maxillary largely developed.

The saliva from this source, like the former, is most abundant while eating, and for a time after. But the flow is easily excited by any movement cf the tongue or jaw; and from close observation I am of the opinion that it is from this gland particularly that we have the responsive reaction against any undue excitements in the buccal cavity, either physical or stimulating.

That from the third or sublingual is least in quantity, being only about $\frac{1}{37}$ of the whole, is still more viscous, thicker and tougher than the others, and is continuously flowing into the mouth, independent of food or motion, moistening and washing the mucous surfaces while the system is fasting or at rest.

These admixed salivas vary in quantity and quality according to the demand and states of the general system and the food partaken of. The quantity is variously estimated at from 8 to 16 oz. in 24 hours, which estimate of course is only approximal. The quality is scarcely ever alike under analysis made at different times, even from the same individual. It has generally, however, an alkaline reaction, varying, it is said, even according to the acidulated state of the food taken, as well as the conditions of the system. Lime enters largely into its composition, soda and potash or their salts, and the phosphates in smaller quantities; animal matter, as ptyalin, fatty acid muous, etc., and water.

The mucous membrane, which has a delicate, velvety surface, is found lining all cavities of the body which have an external communication. Upon and from its surface, is secreted while in a normal state, a tenaceous and viscous mucus, *in quantities only* to keep the parts sufficiently moist and lubricated. This membrane with its exudations is evidently designed to serve as a protecting medium, and of course is found lining the buccal cavity, covering the gums, and in close relation to the teeth. It also contains soda and potash with traces of other earthy matter, also animal matter, as mucin, fat, albumen, etc., together with free acids and water. "Audral contends that pure mucus is always acid in a normal state." This is doubted by others, and perhaps with some propriety, but of this fact we may be certain, that while in an abnormal state it generally shows a decided acid reaction, possessing oftentimes a very irritating effect upon the subjacent tissues, as is often witnessed in a fluent coryza, or a catarrh of the rectum. Lehmann tells us that the secretions separated from the surfaces of the mucous membranes while in a healthy state are absolutely nothing, or nearly so, and any secretions from those surfaces must be regarded as the result of a special or general irritation, and such secretions, if any, are seldom in a normal state, as shown by microscopical and chemical investigations.

When the mouth is closed, the saliva permeates all parts of the buccal cavity, freely commingling with the exudations of the mucous surfaces, neutralizing their acids if any, and even to some extent, perhaps, the free acids found within the cavities of decayed teeth.

But if for the purposes of respiration the mouth is suffered to remain open any length of time, as during rest or sleep, the water of the saliva and mucus is evaporated by the passing current of air. The saliva readily yielding up its alkaline properties to the slightest reaction, immediately parts with it to the carbonic acid of the air, and is thereby rendered useless as a corrective to any acid secretions from the mucous surfaces. While from the evaporation it is thickened near the point of ingress, and its lime uniting with the carbonic acid of the air, discharged from the lungs at each expiration, forms carbonate of lime, and this with the phosphates before existing, and the epithelium and other matters, are deposited upon the teeth, forming the salivary calculi.

During the same time the action of the air irritates the mucous surfaces, stimulating an undue and exhaustive flow of mucus, which from the nature of things must be abnormal and acidulous, that spreads itself over the surfaces of the teeth, parting with its water to the passing current leaves them gummed up with a sordies rendered not only useless, but charged with an acidulous agent highly destructive to their organization; and that, too, owing to the condition of the parts and the nature of things, beyond the neutralizing and restorative functions of the animal organism.

Should it be asked, does the presence of air irritate and inflame mucous surfaces? I should answer, yes; excepting the bronchial and nasal passages, which are differently organized and certainly serve other functions than that intended for the mouth and alimentary canal. All other mucous surfaces are excited when exposed to the air, as witnessed in a prolapsed recti and hemorrhoides. Remove also from the surface of the outer skin (for it also is a mucous membrane) its epidermis and the parts soon become irritated from the presence of the air, which induces an increased flow of mucus, the water of which being evaporated, the remainder forms a scab to shield the delicate membrane from ærial irritation.

Thus we see nature rallying herself to the rescue,—and she does the same to protect the delicate membrane of the mouth. The thickening of the mucus upon the gums and teeth are but the beginnings of a scab, which in time would ultimate in a crust, which sometimes is seen in sickness when the mouth remains open a sufficient length of time; and this crust is to protect the parts from the irritating effects of the air.

'Tis the voice of nature crying aloud *tole causum*, and pointing out to the intelligent observer the evil and its remedy.

